# Serum Concentration of Fluoride in Patients with Alcoholic Liver Cirrhosis from the Lublin Region in Eastern Poland

**DOI:** 10.3390/ijerph18031115

**Published:** 2021-01-27

**Authors:** Andrzej Prystupa, Jarosław Sak, Paweł Kiciński, Agnieszka Stenzel-Bembenek, Anna Błażewicz

**Affiliations:** 1 Department of Internal Medicine, Medical University of Lublin, 20-081 Lublin, Poland; andrzej.prystupa@umlub.pl; 2 Chair and Department of Humanities and Social Medicine, Medical University of Lublin, 20-093 Lublin, Poland; 3 Biobanking and BioMolecular Resources Research Infrastructure, 20-059, Lublin, Poland; 4 Department of Experimental Hematooncology, Medical University of Lublin, 20-093 Lublin, Poland; pawel.kicinski@umlub.pl; 5 Chair and Department of Biochemistry and Molecular Biology, Medical University of Lublin, 20-093 Lublin, Poland; agnieszka.stenzel.bembenek@umlub.pl; 6 Department of Analytical Chemistry, Medical University of Lublin, 20-093 Lublin, Poland; anna.blazewicz@umlub.pl

**Keywords:** alcohol, liver cirrhosis, fluoride, ion chromatography

## Abstract

In view of previous reports, it is important to determine the relationship between liver function and the level of fluoride in the serum. The aim of this study was to investigate serum concentrations of fluoride in 72 patients with alcoholic liver cirrhosis, living in the region of Lublin (Eastern Poland) divided based on the severity of disease according to the Child-Turcotte-Pugh criteria. Higher plasma fluoride concentrations were associated with changes in liver related parameters. In all groups of analyzed patients with different stages of alcoholic liver cirrhosis, elevated levels of plasma fluoride and increased activities of both alanine aminotransferase (ALT) and total bilirubin concentration were shown.

## 1. Introduction

Alcohol-induced liver disease is a common and sometimes fatal consequence of chronic ethanol abuse in many countries and alcohol consumption is considered as the third most significant health risk factor for the global population [1,2] Ethanol metabolism may result in alcohol- induced liver disease, including hepatic steatosis (fatty liver), alcohol-induced hepatitis, cirrhosis and cancer. The main toxic products in ethanol metabolism include acetaldehyde and free radicals. At the beginning, the liver injury can be reversible: the liver is enlarged, full of fat with collagen fibers, but still has regenerative potential and activity. Then, changes become irreversible—destruction of normal liver architecture and decreased blood flow cause loss of the liver function and hepatic failure (cirrhosis). As a consequence, in patients with alcohol-induced liver disease (ALD) inappropriate levels of drugs, xenobiotics and heavy metals are often observed, compared to healthy individuals [3,4,5]. Alcoholic cirrhosis is defined by the occurrence of extensive fibrosis and regenerative nodules. The Model of End-Stage Liver Disease (MELD) and Child-Pugh Score are used to assess the severity of liver disease and timing for liver transplantation [6].

Fluoride (F) is a trace element which is naturally present in soil, water and food and is also known as an important environmental toxicant, obtained from industrial sources. Fluorides are normally used in low concentrations to reduce the incidence of caries and proper bone mineralization, whereas chronic excessive fluoride exposure, resulting in higher blood concentration and accumulation in different tissues, due to problems with its metabolism and body utilization can impair human health and even induce fluorosis. Chronic excessive fluoride exposure impairs human health and damages the skeletal system and teeth. This leads to simultaneous increase of osteocalcin and calcitonin levels in plasma which may cause fluoride-dependent bone damage and dental fluorosis, characterized by hypo-mineralization of enamel and dentine. Because of the high fluoride affinity to calcium ions, calcification of tissues, vascular walls, synovial capsules and ligaments appears, leading to persistent joint pain and limited join movement [7,8]. Studies based on animal models have shown oxidative stress, DNA damage and apoptosis in liver as an effect of fluorosis [9,10,11]. However, the mechanism of fluoride-induced hepatic toxicity has not been definitely explained. Zhao et al. suggested that fluoride induced apoptosis and autophagy in liver was caused by activating the IL-17 signaling pathway [12]. Girardi and Merler indicated that a high cumulative internal dose of perfluorooctanoic acid showed a statistically significant increase for mortality from liver cancer and liver cirrhosis in male employees [13]. In view of the previous reports, it is important to determine the relationship between stages of alcoholic liver cirrhosis and the level of fluoride in the serum. The aim of this study was to investigate serum concentrations of fluoride in patients with alcoholic liver cirrhosis living in the region of Lublin (Eastern Poland) according to different cirrhosis stages.

## 2. Experimental Section

### 2.1. Patients

The study was conducted at the Department of Internal Medicine, Medical University of Lublin, Poland, and included 72 patients with alcoholic liver cirrhosis from the region of Lublin, (Eastern Poland). The study protocol was approved by the Bioethics Committee at the Medical University of Lublin, Poland (agreement number KE-0254/349/2015).

All subjects gave their written informed consent for participation in the study. According to the World Health Organization (WHO) the optimal fluoride concentration recommended in drinking water normally ranges between 0.5 and 1 mg/L and accounts for approximately 40–70% of daily fluoride ingestion. In the Eastern Poland region, water fluoride concentration was estimated by the Municipal Water and Sewerage Company in Lublin (MPWiK) at around ≤0.5 mg/L [14].

Liver cirrhosis was diagnosed based on clinical features, history of heavy alcohol consumption, laboratory tests and abdominal ultrasonography. Patients with alcoholic hepatitis, hepatocellular carcinoma, viral and autoimmune diseases were excluded from the study. Other exclusion criteria were type 2 diabetes, obesity, acute infections (e.g., pneumonia, spontaneous bacterial peritonitis), acute and chronic heart failure the New York Heart Association classification (>NYHA I), acute and chronic respiratory disorders resulting in respiratory insufficiency, acute kidney injury (AKI) and chronic kidney disease (CKD > stage G2), and excessive exposure in the workplace or living environment to fluoride. Both clinical assessment and laboratory tests were used to exclude underlying liver diseases in the control group. The degree of liver cirrhosis was evaluated according to the Pugh-Child criteria (Pugh-Child score). Based on this, patients were assigned to one of three groups: Pugh-Child (P-Ch) A—21 with stage A, P-Ch B—23 with stage B and P-Ch C—28 with stage C liver cirrhosis. The control group consisted of 22 healthy individuals without liver disease who did not abuse alcohol. Clinical, biochemical and demographic detailed characteristics of patients are presented in Table 1 and Table 2.

### 2.2. Instrumentation and Reagents

The determination of fluoride in studied samples was carried out by ion chromatography (IC) with a suppressed conductometric detection (Dionex DX 500 system consisting of: GP40 Gradient Pump, CD20 Conductivity Detector, and Chromeleon Chromatography Workstation, Dionex, Sunnyvale, CA, USA). The IC method is routinely used in the analysis of fluorides in waters (Chemical Suppression of Eluent Conductivity Method (Environmental Protection Agency—EPA 300.0, ASTM D4327-91 and Standard Methods 4110B, ISO 10359-1); however, plasma samples present a complicated matrix for IC (samples which contain organic material are capable of fouling an ion-exchange column). Therefore, before the analysis each sample underwent an acetonitrile deproteinization procedure (0.5 mL of serum was mixed with an equal volume of acetonitrile, then centrifuged at 5000× *g* for 8 min. Then 0.1 mL of the supernatant was diluted with 1.0 mL deionized water (resistivity of 18.18 mΩ·cm at 25 °C). Samples of serum were spiked with fluoride standard solution, at concentration levels of 0.5, 1.0, and 2.0 ppm (recoveries ranged between 96.9–99.0%). Stock standard solution (1000 mg/L) of fluoride was prepared by dissolving an appropriate amount of analytical reagent grade sodium salt (Merck (Darmstadt, Germany)) in deionized water.

Chromatographic determinations were carried out according to the manufacturer’s guidelines with the use of IonPac^®^AS22 Analytical and IonPac AG22 Guard columns dedicated to the analysis of inorganic anions. A mobile phase consisted of 4.5 mMNaHCO_3_ and 1.4 mM Na_2_CO_3_. Flow rate of 1.2 mL/min and injection volume of 25 µL were applied.

Linearity was in the range of 0.01–2.0 mg/L with a correlation coefficient of 0.9996. The limit of detection (LOD, S/N = 3) and limit of quantification (LOQ, S/N = 10) were 3.80 µg/L and 11.40 µg/L, respectively. The relative standard deviations of peak area and peak height were all less than 6.56%.

### 2.3. Statistical Analysis

STATISTICA 13.3 (TIBCO Software Inc., Palo Alto, CA, USA) was used for data analysis. Continuous variables were expressed as the mean ± standard deviation (SD). Before calculations, variables were checked for normality using the Shapiro-Wilk test. The Levene’s test was applied to test equality of variances. To compare the results between more than two groups, one-way ANOVA test was used. The Scheffe’s test was applied for post hoc analysis. Correlations among variables were performed using the Pearson’s correlation test. Qualitative variables are shown as indicators of structure (percentage); for intergroup comparisons the χ^2^ test was used. For all tests, *p* < 0.05 was considered as statistically significant.

## 3. Results

The clinical and demographic characteristics of the control and alcoholic liver cirrhosis groups are presented in Table 1 and Table 2.

**Figure 1:**
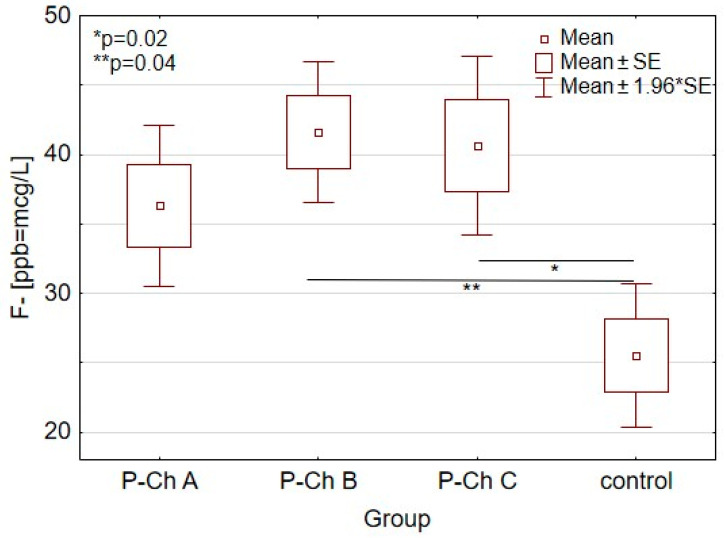
Serum fluoride concentrations in patients with Alcoholic Liver Cirrhosis. *: *p* = 0.02, **: *p* = 0.04.

The study findings demonstrated that the serum fluoride level was least statistically significant in the control group: 25.5 ± 17.7 µg/L (ppb) (Table 3 and Figure 1). The serum fluoride concentration increased in the subsequent stages of alcoholic liver cirrhosis to 36.3 ± 10.2 µg/L in P-Ch A, to 41.6 ± 15.6 µg/L in P-Ch B and to 40.6 ± 20.5 µg/L in P-Ch C. Statistically significantly differences in this aspect were indicated between the control group and the subgroup of P-Ch B (*p* = 0.02) and the subgroup of P-Ch C (*p* = 0.04).

Correlations between serum fluoride concentrations versus selected laboratory parameters were also analyzed. Positive correlations were found between level of serum fluoride versus the level of total bilirubin (r = 0.21; *p* = 0.02), mean cell volume (MCV) (r = 0.25; *p* = 0.01) and ALT (r = 0.19; *p* = 0.045) (Table 4).

There were no statistically significant differences in serum fluoride level depending on sex (*p* = 0.74). The presence of complications from liver cirrhosis was not associated with significant differences in the concentration of fluoride (*p* = 0.49 in the case of ascites, *p* = 0.31 in the case of encephalopathy, or *p* = 0.21 in the case of esophageal varices).

## 4. Discussion

Fluoride absorption is mainly due to passive diffusion without any specific transporters, however, recently protein-based channels in phospholipid membranes with high selectivity for F^-^ were found, but their role for mammals is still not clear [15,16]. Insoluble complexes formed by fluoride and Ca^+2^, Mg^+2^, Al^+3^ obtained from the food can decrease fluoride absorption, which explains the treatment of acute fluoride toxicity using calcium-containing solutions [17]; in contrast, the addition of phosphates, sulfates and molybdenum increases its absorption [18]. Following absorption from the stomach and small intestine, approximately 50% of the absorbed F^-^ is quickly incorporated in calcified tissues—mainly in teeth and bones, as fluoro-hydroxyapatite, where it increases osteoblast proliferation and stimulates bone formation. A minor portion of fluoride is absorbed by soft tissues like kidney, liver, lung, muscle, spleen, reproductive and endocrine glands. Non-absorbed fluoride is rapidly removed through renal excretion and excreted in feces [19,20].

It is known that proper kidney glomerular filtration and excretion means a proper plasma fluoride level and different factors causing disturbances in the acid-base balance such as metabolic disorders, respiratory diseases, drugs, toxic substances, and physical activity can change this situation. In this case urine pH is also changed which creates a different level of ionic fluoride F—reabsorption back to systemic circulation from 10% up to 90%. [20,21].

The stage of liver cirrhosis correlates with failure of kidney function. This may show the hepatorenal syndrome form that is difficult to diagnose in the early stage. For this reason, creatinine level is not a good indicator of kidney function in these patients. Yoo et al. indicate that reduced muscle mass has a great impact on overestimation of kidney function in patients with cirrhosis [22]. In order to eliminate the influence of renal dysfunction on the level of fluoride in the blood, we excluded patients with chronic kidney disease (CKD > G2) and acute kidney disease (AKI) from the study.

Fluoride may be significantly dangerous to the human nervous system and have been known to penetrate the blood-brain barrier, where, through generation of free radicals, oxidative stress conditions are promoted, causing impaired function of myelin, neurons and neurotransmitters, associated with lower IQ scores, depression, disturbances in learning processes and psychomotor skills [7,23,24,25]. Several studies have reported that fluoride can be toxic to the reproductive system causing decreased fertility [26,27] Recently, reports have indicated fluoride toxicity during immune and inflammatory responses and impaired glucose tolerance with insulin resistance in peripheral tissues [7]. Stomach pains, loss of appetite, polyuria, polydipsia, muscle weakness, and constipation followed by diarrhea are found to be important diagnostic criteria of non-skeletal fluorosis [28].

In our study higher serum fluoride concentrations were associated with changes in liver related parameters. In all Pugh-Child score groups of analyzed patients with different stages of alcoholic liver cirrhosis, elevated levels of plasma fluoride, increased activities of both hepatic aminotransferases, ALT and AST, and total bilirubin concentration were shown. The risk of serious complications like esophageal varices, ascites and encephalopathy was highest in the most advanced stage of cirrhosis (P-Ch C), which determines that these two toxic agents, alcohol and fluoride, might act synergistically in the liver damage process. The time of alcohol consumption in this group was also the longest.

Blood fluoride content (serum fluoride level) can reflect the external environmental exposure level of an organism to this element. In our study all patients (control and study groups) were from the Eastern region of Poland where the drinking water fluoride concentration range was below 0.5 mg/L (below 500 ppb) in the period January 2018–December 2019, according to information given by the Municipal Water and Sewerage Company in Lublin (MPWiK) responsible for communal water distribution in the region [14]. However, when designing nationwide population biobanking for scientific studies, the possibility of the common determination of heavy metals and fluoride in the serum of biobank’s blood donors should be considered as a reliable assessment of environmental exposure [29,30].

Taves indicated that the average serum fluoride concentration of sixteen individuals was 0.013 ppm (13 ppb) [31]. In our study, this level was 0.0255 ± 0.0177 ppm (25.5 ± 17.7 ppb) in the control group. According to Singer and Armstrong there was plasma fluoride content within the range 0.14–0.26 ppm (140–260 ppb) [32]. They conducted their research in three American States (Minnesota, Michigan, South Dakota) in the period 1958–1959 where fluoride levels in water were in the range from 0.15 to 5.4 ppm (150–5400 ppb). The highest fluoride level was observed among individuals from Lake Preston (South Dakota)—0.26 ± 0.0124 ppm (260 ± 12.4 ppb), where the fluoride level of communal showed the highest level at 5.4 ppm (5400 ppb) [32]. It is worth noticing that the average plasma fluoride level which observed in Singer and Armstrong’s study in this American region was ten times higher than the average level in our study’s control group. However, the level of fluoride in communal water in Lake Preston fifty years ago was also more than ten times higher than the municipal water that patients drank in the Lublin region in 2018–2019 [14].

The main source of fluoride is water. However, it can be found in other beverages and foods. Alcoholic beverages can be an important source of fluoride, especially in the group of ALD patients. Paz et al. identified the fluoride concentration of 53 samples of organic and non-organic wines (the Canary Islands and mainland Spain) within the range of 0.03 to 0.70 mg/L [33]. In a previous study, two decades earlier, Martinez et al. stated that the mean concentration of fluoride in 70 samples of wine (the Canary Islands) from a region with a high concentration of fluoride in drinking water was significantly higher than the mean concentration in other samples [34]. In the Lublin region, beer is consumed much more often than wine. Therefore, the research carried out by Styburski et al. was important [35]. They compared the fluoride concentration in different beer samples: Thailand (0.260 ppm), Italy (0.238 ppm), Mexico (0.210 ppm), China (0.203 ppm) and Polish beers (0.089 ppm). Goschorska et al. compared the fluoride concentration in 48 types of drink with low, medium, and high alcohol content available in Poland, both Polish and foreign [36]. They stated that the highest fluoride levels were determined in beers and wines, while the lowest levels were identified in vodkas.

The production of ethanol oxidation-acetaldehyde can enhance free-radical damage by binding to glutathione and other free-radical defense enzymes in liver tissue. Fluoride is also known to destroy biomolecules through generation of reactive oxygen species (ROS) and to augment the oxidative stress condition due to inhibition or interaction with antioxidative enzymes which makes the liver tissue more susceptible to biochemical injury by other toxicant molecules [37].

Recent evidence has reported that fluoride can also augment the oxidative stress condition in kidneys by increasing the concentration of ROS in kidney tissue with decreased levels of glutathione (GSH), accompanied by decreased GST activity in kidney tissue [37]. Once again, the imbalanced pro-oxidant/antioxidant status, leading to the generation of oxidative stress conditions, is shown as a result of fluoride toxicity. The results suggest that higher circulating fluoride levels are the results of liver dysfunction. However, it would be worth assessing the content of fluoride in liver biopsies in further research.

Our study has some limitations: a small number of subjects and voluntary selection, difficulties in assessing actual renal function, no assessment of fluoride concentration in liver bioptates and omitting, in the protocol of our study, the consumption of alcoholic beverages (amount and type of drink).

However, this is the first attempt to assess the relationship between serum concentration of fluoride and the stages in alcoholic liver cirrhosis. We tried to show that higher serum fluoride concentrations are associated with changes in liver related parameters and stages of cirrhosis. This study contributes to further research, e.g., observational, multi-center studies on the relationship between serum concentration of fluoride and the development of ALD and the consumption of alcoholic beverages. We also suggest the need of experimental studies to elucidate the molecular mechanisms of fluoride during ALD.

## Figures and Tables

**Table 1:** Demographic and clinical characteristics of the study and control groups (mean ± SD).

Characteristics	Control Group	Alcoholic Liver Cirrhosis		
		Pugh-Chile (P-Ch) A	P-Ch B	P-Ch C
Age (years)	44.6 ± 15.9 *	55.2 ± 12.6 *	54.4 ± 11.5 *	58.5 ± 7.6 *
Male (%)	64.3%	75%	67.5%	61.5%
Body mass (kg)	68.1 ± 9.8	68.4 ± 14.6	69.7 ± 11.9	70.8 ± 12.5
Time of alcohol abuse (years)	N/A	12.9 ± 4.8 ^†^	14.6 ± 5.1	15.8 ± 5.5 ^†^
Complications:				
Esophageal varices	N/A	29.6% ^†,¥^	65% ^†,#^	87.2% ^¥,#^
Ascites	N/A	22.2% ^†,¥^	48.7% ^†,#^	92.1% ^¥,#^
Encephalopathy	N/A	18.5% ^†,¥^	51.3% ^†,#^	84.6% ^¥,#^

* *p* < 0.05—statistically significant differences between control group and P-Ch subgroups; ^†,¥,#,-^ *p* < 0.05 statistically significant differences between P-Ch subgroups.

**Table 2:** Biochemical characteristics of the study and control groups (mean ± SD).

Variables	Control Group	Alcoholic Liver Cirrhosis		
		P-Ch A	P-Ch B	P-Ch C
Total bilirubin (mg/dL)	0.55 ± 0.28 *	3.9 ± 8.3 *^,†^	3.8 ± 2.9 *^,¥^	8.2 ± 8.8 *^,†,¥^
Albumin (g/dL)	-	3.25 ± 0.81 ^†^	2.9 ± 0.39	2.49 ± 0.51 ^†^
Total protein (g/dL)	6.3 ± 0.3	6.1 ± 0.9	5.9 ± 1	5.8 ± 0.9
Blood platelets (g/L)	235.7 ± 31.5 *	183.8 ± 77.4 ^†,¥^	138.1 ± 76.9 *^,†^	142 ± 77.7 *^,†,¥^
Mean Cell Volume (MCV) (fl)	84.8 ± 3.8 *	91.3 ± 7.2	91.6 ± 10.9	95.8 ± 7.5 *
INR	-	1.26 ± 0.33 ^†,¥^	1.45 ± 0.22 ^†^	1.55 ± 0.34 ^¥^
ALT (U/L)	18.1 ± 6.8 *	92.1 ± 189.8 *^,†,¥^	36.1 ± 25 *^,†,¥^	41.7 ± 29.5 *^,†,¥^
AST (U/L)	18.1 ± 7.1 *	123.1 ± 202.3 *^,†,¥^	82.3 ± 65.9 *^,†,¥^	90.6 ± 62.4 *^,†,¥^
Urea (mg/dL)	-	33.3 ± 19.9 ^†,¥^	22.6 ± 15.8 ^†,#^	42.7 ± 35.9 ^¥,#^
Sodium (mmol/L)	139.8 ± 3.7	133.7 ± 5.7	135 ± 3.4	134.1 ± 5.7
Potassium (mmol/L)	4.44 ± 0.42 *	4 ± 0.6 *	4 ± 0.63 *	3.9 ± 0.6 *
C-reactive protein (mg/L)	2.17 ± 1.86 *	14.7 ± 17.2 *^,†,¥^	29.2 ± 51.6 *^,†^	25.7 ± 20.1 *^,¥^

* *p* < 0.05—statistically significant differences between control group and P-Ch subgroups; ^†,¥,#,-^ *p* < 0.05 statistically significant differences between P-Ch subgroups.

**Table 3:** Serum fluoride concentrations in Patients with Alcoholic Liver Cirrhosis Compared to the Control Group.

	Control Group (*n* = 15)	Alcoholic Liver Cirrhosis (*n* = 72)			*p*
		P-Ch A (*n* = 21)	P-Ch B (*n* = 23)	P-Ch C (*n* = 28)	
(F-[ppb = µg/L)	25.5 ± 17.7 *	36.3 ± 10.2	41.6 ± 15.6 *	40.6 ± 20.5 *	0.01

* *p* < 0.05—statistically significant differences between control group and P-Ch subgroups.

**Table 4:** Correlation between serum fluoride concentration and total bilirubin, MCV, ALT.

Variables	Correlation Coefficient *p*-Value	
**Fluoride [ppb = µg/L]**	Total bilirubin (mg/dL)	r = 0.21
		*p* = 0.02
	Mean cell volume (MCV)	r = 0.25
		*p* = 0.01
	Alanine aminotransferase (ALT)	r = 0.19
		*p* = 0.045

## Data Availability

The data presented in this study are available on request from the corresponding author.

## References

[1] Adiamah A., Ban L., Hammond J., Jepsen P., West J., Humes D.J. (2020). Mortality after extrahepatic gastrointestinal and abdominal wall surgery in patients with alcoholic liver disease: A systematic review and meta-analysis. Alcohol Alcohol..

[2] Zatonski W.A., Sulkowska U., Manczuk M., Rehm J., Boffetta P., Lowenfels A.B., La Vecchia C. (2010). Liver cirrhosis mortality in Europe, with special attention to Central and Eastern Europe. Eur. Addict Res..

[3] Whitford G.M., Pashley D.H., Reynolds K.E. (1979). Fluoride tissue distribution: Short-term kinetics. Am. J. Physiol..

[4] Prystupa A., Szpetnar M., Boguszewska-Czubara A., Grzybowski A., Sak J., Załuska W. (2015). Activity of MMP1 and MMP13 and amino acid metabolism in patients with alcoholic liver cirrhosis. Med. Sci. Monit..

[5] Prystupa A., Błażewicz A., Kiciński P., Sak J.J., Niedziałek J., Załuska W. (2016). Serum concentrations of selected heavy metals in patients with alcoholic liver cirrhosis from the Lublin region in Eastern Poland. Int. J. Environ. Res. Public Health.

[6] European Association for the Study of Liver (2012). EASL clinical practical guidelines: Management of alcoholic liver disease. J. Hepatol..

[7] Zuo H., Chen L., Kong M., Qiu L., Lü P., Wu P., Yang Y., Chen K. (2018). Toxic effects of fluoride on organisms. Life Sci..

[8] Błaszczyk I., Birkner E., Gutowska I., Romuk E., Chlubek D. (2012). Influence of methionine and vitamin E on fluoride concentration in bones and teeth of rats exposed to sodium fluoride in drinking water. Biol. Trace Elem. Res..

[9] Niu Q., He P., Xu S., Ma R., Ding Y., Mu L., Li S. (2018). Fluoride-induced iron overload contributes to hepatic oxidative damage in mouse and the protective role of grape seed proanthocyanidin extract. J. Toxicol. Sci..

[10] Liang C., Gao Y., Zhao Y., Manthari R.K., Ma J., Niu R., Wang J., Zhang J., Wang J. (2018). Effects of fluoride and/or sulfur dioxide on morphology and DNA integrity in rats’ hepatic tissue. Biol. Trace Elem. Res..

[11] Lu Y., Luo Q., Cui H., Deng H., Kuang P., Liu H., Fang J., Zuo Z., Deng J., Li Y. (2017). Sodium fluoride causes oxidative stress and apoptosis in the mouse liver. Aging.

[12] Zhao Y., Li Y., Wang J., Manthari R.K., Wang J. (2018). Fluoride induces apoptosis and autophagy through the IL-17 signaling pathway in mice hepatocytes. Arch. Toxicol..

[13] Girardi P., Merler E. (2019). A mortality study on male subjects exposed to polyfluoroalkyl acids with high internal dose of perfluorooctanoic acid. Environ. Res..

[14] (2020). Municipal Water and Sewerage Company in Lublin [Miejskie Przedsiębiorstwo Wodociągów i Kanalizacji w Lublinie Sp. z o.o. (MPWiK)]. http://www.mpwik.lublin.pl/index.php?option=site&id=5&sid=24.

[15] Stockbridge R.B., Lim H.H., Otten R., Williams C., Shane T., Weinberg Z., Miller C. (2012). Fluoride resistance and transport by ribiswitch-controlled CLC antiporters. Proc. Natl. Acad. Sci. USA.

[16] Zohoori F.V., Innerd A., Azevedo L.B., Whitford G.M., Maguire A. (2015). Effect of exercise on fluoride metabolism in adult humans: A pilot study. Sci. Rep..

[17] Li L. (2003). The biochemistry and physiology of metallic fluoride: Action, mechanism and implications. Crit. Rev. Oral. Biol. Med..

[18] Gazzano E., Bergandi L., Riganti C., Aldieri E., Doublier S., Costamagna C., Bosia A., Ghigo D. (2010). Fluoride effects: The two faces of janus. Curr. Med. Chem..

[19] Buzalaf M.A.R., Whitford G.M. (2011). Fluoride metabolism. Monogr. Oral. Sci..

[20] Buzalaf C.P., de L. Leite A., Buzalaf M.A.R., Preedy V.R. (2015). Fluoride Metabolism. Fluorine: Chemistry, Analysis, Function and Effects.

[21] Sharma R., Tsuchiya M., Skobe Z., Tannous B.A., Bartlett J.D. (2010). The acid test of fluoride: How pH modulates toxicity. PLoS ONE.

[22] Yoo J.J., Kim S.G., Kim Y.S., Lee B., Lee M.H., Jeong S.W., Jang J.Y., Lee S.H., Kim H.S., Kim Y.D. (2019). Estimation of renal function in patients with liver cirrhosis: Impact of muscle mass and sex. J. Hepatol..

[23] Kupnicka P., Listos J., Tarnowski M., Kolasa-Wołosiuk A., Wąsik A., Łukomska A., Barczak K., Gutowska I., Chlubek D., Baranowska-Bosiacka I. (2020). Fluoride affects dopamine metabolism and causes changes in the expression of dopamine receptors (D1R and D2R) in chosen brain structures of morphine-dependent rats. Int. J. Mol. Sci..

[24] Saeed M., Malik R.N., Kamal A. (2020). Fluorosis and cognitive development among children (6–14 years of age) in the endemic areas of the world: A review and critical analysis. Environ. Sci. Pollut. Res..

[25] Riddell J.K., Malin A.J., Flora D., McCague H., Till C. (2019). Association of water fluoride and urinary fluoride concentrations with attention deficit hyperactivity disorder in Canadian youth. Environ. Int..

[26] Zhang S., Jiang C., Liu H., Guan Z., Zeng Q., Zhang C., Lei R., Xia T., Gao H., Yang L. (2013). Fluoride-elicited developmental testicular toxicity in rats: Roles of endoplasmic reticulum stress and inflammatory response. Toxicol. Appl. Pharmacol..

[27] Ma Q., Huang H., Sun L., Zhou T., Zhu J., Cheng X., Duan L., Li Z., Cui L., Ba Y. (2017). Gene-environment interaction: Does fluoride influence the reproductive hormones in male farmers modified by ERα gene polymorphisms?. Chemosphere.

[28] Pramanik S., Saha D. (2017). The genetic influence in fluorosis. Environ. Toxicol. Pharmacol..

[29] Sak J., Pawlikowski J., Goniewicz M., Witt M. (2012). Population biobanking in selected European countries and proposed model for a Polish national DNA bank. J. Appl. Genet..

[30] Witoń M., Strapagiel D., Gleńska-Olender J., Chróścicka A., Ferdyn K., Skokowski J., Kalinowski L., Pawlikowski J., Marciniak B., Pasterk M. (2017). Organization of BBMRI.pl: The Polish biobanking network. Biopreserv Biobank..

[31] Taves D.R. (1966). Normal human serum fluoride concentrations. Nature.

[32] Singer L., Armstrong W.D. (1960). Regulation of human plasma fluoride concentration. J. App. Physiol..

[33] Paz S., Jaudenes J.R., Gutiérrez A.J., Rubio C., Hardisson A., Revert C. (2017). Determination of fluoride in organic and non-organic wines. Biol. Trace Elem. Res..

[34] Martínez O.B., Díaz C., Borges T.M., Díaz E., Pérez J.P. (1998). Concentrations of fluoride in wines from the Canary Islands. Food Addit. Contam..

[35] Styburski D., Baranowska-Bosiacka I., Goschorska M., Chlubek D., Gutowska I. (2017). Beer as a rich source of fluoride delivered into the body. Biol. Trace Elem. Res..

[36] Goschorska M., Gutowska I., Baranowska-Bosiacka I., Rać M.E., Chlubek D. (2016). Fluoride content in alcoholic drinks. Biol. Trace Elem. Res..

[37] Shanthakumari D., Srinivasalu S., Subramanian S. (2004). Effects of fluoride intoxication on lipidperoxidation and antioxidant status in experimental rats. Toxicology.

